# Effect of adenotonsillectomy on the growth, development, and comprehensive cognitive abilities of children with obstructive sleep apnea: a prospective single-arm study

**DOI:** 10.1186/s12887-022-03111-w

**Published:** 2022-01-15

**Authors:** Shan Shan, Shuyu Wang, Xue Yang, Fan Liu, Linying Xiu

**Affiliations:** 1 Department of Otolaryngology Head and Neck Surgery, The No 980 Hospital, Joint Logistics Support Force, PLA, Shijiazhuang, Hebei Province China; 2 Department of Pediatrics, The No 980 Hospital, Joint Logistics Support Force, PLA, Shijiazhuang, Hebei Province China; 3 Department of Otolaryngology Head and Neck Surgery, Handan Central Hospital, Handan, Hebei Province China

**Keywords:** Obstructive sleep apnea, Children, Adenotonsillectomy, Growth, Development, Cognitive abilities

## Abstract

**Background:**

Previous studies did not comprehensively examine the effect of adenotonsillectomy on growth and development, emotional state, quality of life, attention ability, and cognitive dysfunction in children with obstructive sleep apnea (OSA). This study aimed to explore the improvement effects of adenotonsillectomy on the growth, development, quality of life, and attention ability in children with OSA.

**Methods:**

This prospective single-arm study involved children with OSA admitted at The No. 980 Hospital, Joint Logistics Support Force, PLA, China (02/2017–02/2018). The Myklebust Pupil Rating Scale (PRS), Inventory of Subjective Life Quality (ISLQ), Zung Self-rating Anxiety Scale (SAS), Conners Parent Symptom Questionnaire (PSQ), and Continuous Performance Task (CPT) were examined before and at 6 months after adenotonsillectomy.

**Results:**

Forty-nine patients were enrolled. They all completed the 6-month follow-up. The body mass index increased after surgery (from 18.8 ± 4.9 to 19.3 ± 4.3 kg/m^2^, *P* = 0.008). The total PRS score increased 6 months after surgery (from 73.8 ± 12.7 to 84.6 ± 10.3, *P* < 0.001). All aspects of the ISLQ, except anxiety experience and physical emotion, were improved at 6 months after adenotonsillectomy (all *P* < 0.01). The SAS score also decreased from 20.1 ± 10.0 to 12.8 ± 6.6 (*P* < 0.001). All six dimensions of the PSQ, as assessed by the legal guardians, decreased after adenotonsillectomy (all *P* < 0.01). The proportions of children with auditory and/or visual sustained attention abnormalities decreased after surgery.

**Conclusions:**

After adenotonsillectomy, the PRS, ISLQ, and PSQ improved, and anxiety and auditory/visual sustained attention abnormalities decreased, suggesting positive impacts on the growth, development, quality of life, and comprehensive cognitive abilities of children with OSA.

## Background

Obstructive sleep apnea (OSA) is a breathing disorder characterized by recurrent complete or partial upper airway obstruction during sleep [1]. OSA interrupts the normal ventilation in sleep resulting in intermittent hypoxia and hypercapnia, frequent arousals, and sleep fragmentation. In addition, OSA may result in chronic snoring symptoms (commonly with sporadic pauses, snorts, or gasps), disturbed sleep patterns, and daytime neurobehavioral problems [1]. Apnea is defined as mouth and nose airflow decreased by > 90% for at least two breathing cycles, despite chest and abdomen movements during the event [2]. Hypopnea is defined as mouth and nose airflow decreased by > 30% compared with baseline, lasting for at least two breathing cycles, with event-related waking up or a decrease of ≥3% in oxygen saturation [2]. OSA is mild if 1/h < OAHI≤5/h, moderate if 5/h < OAHI≤10/h, or severe if OAHI> 10/h [1]. The prevalence of OSA is 1–6% in children [1, 3–6]. Interventions for OSA in children include intranasal steroids [7, 8], montelukast [9], and continuous positive airway pressure (CPAP), but the only curative and definitive treatment is adenotonsillectomy [10, 11].

The complications of OSA in children include physiological and physical complications including impaired cardiac function (pulmonary arterial hypertension, heart dilation, chronic cardiac dysfunction, and increased in right heart afterload) [12, 13], impaired growth and development [14], and failure to thrive [15]. In addition to physical complications, OSA has detrimental effects on behavior and cognition [16, 17], development [14], emotional state [18, 19], quality of life [18, 20, 21], attention ability [19, 22], and cognitive function [19, 23, 24].

Still, although adenotonsillectomy can cure OSA [10, 11] and improve diastolic function [25, 26], the previous studies on the impact of adenotonsillectomy on cognition and behavior yielded conflicting results. Indeed, Marcus et al. [11] reported that adenotonsillectomy improves the quality of life, impulsiveness, and emotional lability, but not attention and cognitive functions. A Cochrane review of only three studies suggested that evidence is lacking regarding the impact of adenotonsillectomy on quality of life, symptoms, and behavior [27, 28]. Of note, the available studies did not comprehensively examine the effect of adenotonsillectomy on growth and development, emotional state, quality of life, attention ability, and cognitive dysfunction. Furthermore, the previous studies were performed in Western settings, and very little data are available for Chinese children.

Therefore, this prospective study aimed to explore the improvement effects of adenotonsillectomy on the growth, development, quality of life, and attention ability in Chinese children with OSA.

## Methods

### Study design and participants

This prospective single-arm study involved children with OSA admitted to the Department of Otolaryngology at The No. 980 Hospital, Joint Logistics Support Force, PLA, China, from February 2017 to February 2018.

The diagnostic criteria for obstructive sleep apnea in children were based on the third edition of the International Classification of Sleep Disorders (ICSD-3) developed by the American Academy of Sleep Medicine (AASM) [29]. The inclusion criteria were 1) 6–12 years of age, 2) > 3-month history of sleep snoring and mouth breathing, 3) consistent with the indications of adenotonsillar surgery (a. chronic tonsillitis occurs repeatedly and acutely, b. excessive tonsillitis hinders swallowing, breathing, and vocal function, c. chronic tonsillitis has become a “focus” causing lesions of other organs or is associated with lesions of adjacent organs, d. tonsillar hypertrophy leads to obstructive sleep apnea, e. drug treatment for adenoidal hypertrophy is ineffective, f. Severe adenoid hypertrophy, g complications such as otitis media, sinusitis, and posterior nostril drip, h. occlusive nasal sound, i. adenoid face, j. weight loss and developmental disorder), and 4) diagnosis of OSA was confirmed by PSG. The exclusion criteria were 1) central sleep apnea syndrome or hypopnea syndrome, 2) OSA complicated with other diseases (such as Down syndrome, severe craniofacial deformities, neuromuscular disorders, chronic lung diseases, sickle cell disease, metabolic diseases, or laryngomalacia), 3) refusal to undergo surgical intervention, or 4) surgical contraindications (including acute tonsillitis attack period, diseases of the hematopoietic system, hypocoagulation, or severe systemic diseases).

This study was approved by the Ethics Committee of The No. 980 Hospital, Joint Logistics Support Force, PLA, China (2021-KY-2). All patients’ guardians provided written informed consent.

### Outcome measures

Baseline data included age, sex, height, weight, body mass index (BMI), learning disabilities, quality of life, anxiety, behavioral problems, and attention ability.

Considering that all the methods used in this study were non-invasive and to avoid subjective bias as much as possible, we only informed the children and their parents of the cognitive and developmental results of this study and explained the reasons to the parents after the evaluation at 6 months postoperatively. In addition, the parents could withdraw at any time if they did no longer agree to participate in the study.

### Questionnaires and test methods

The revised Myklebust Pupil rating scale (PRS) was used for screening for learning disabilities. The PRS evaluates auditory comprehension, spoken language, orientation, motor coordination, and personal-social behavior. It is summarized as verbal subtotal, nonverbal subtotal, and total scores. The Chinese version of the PRS has coefficients of reliability > 0.95 for both subscores [30].

The Inventory of subjective life quality (ISLQ) includes 52 items that assess family life, peer relationship, school life, living environment, self-knowledge, the cognitive component, the experience of depression, the experience of anxiety, physical emotion, and emotional component [31]. It has been validated in Chinese, and Cronbach’s α is 0.89 [32].

The Zung Self-Rating Anxiety Scale (SAS)-CR is a widely used tool for screening anxiety based on manifestations in the cognitive, autonomic, motor, and central nervous system dimensions [33]. It has been validated in Chinese, with a Cronbach’s α of 0.931 [34].

Conners parent symptom questionnaire (PSQ) evaluates behavioral markers that include hyperactivity, compulsive actions, perfectionism, playing up in class, violence, aggressiveness, mathematics difficulties, language difficulties, fear of separation, social issues, and emotional anguish [35]. It is validated in Chinese and has a Cronbach’s α > 0.85 [36].

Attention ability (visual and/or auditory) was assessed by the Continuous Performance Task (CPT), which included sustained attention abnormalities of visual sense, auditory, and both visual and auditory [37].

Overnight PSG monitoring was performed using Condi E polysomnography equipment. Electroencephalogram, electrooculogram, chin electromyography, electromyography of the lower limbs (leg movement sensor), respiratory airflow (oral-nasal temperature airflow sensor, nasal pressure sensor), respiratory effort (chest and abdominal band), blood oxygen saturation (pulse oxygen meter), body position, snoring, electrocardiograph, and real-time digital video were recorded synchronically.

### Surgical intervention and follow-up

Adenotonsillectomy was performed by one of three senior deputy doctors with over 10 years of relevant surgical experience. The above questionnaires and tests were evaluated in the inpatient department before surgery and in the outpatient department 6 months after surgery.

### Statistical analysis

All data were analyzed using SPSS 22.0 (IBM, Armonk, NY, USA). The Kolmogorov-Smirnov normality test was performed on all continuous variables. Normally distributed continuous variables were presented as means ± standard deviations and analyzed using paired t-test. Non-normally distributed continuous variables were presented as medians (interquartile ranges) and analyzed using Wilcoxon’s test. The categorical data were presented as n (%) and analyzed using the McNemanr test for non-ranked data and Wilcoxon’s test for ranked data. All statistical tests were two-tailed, and *P*-values < 0.05 were considered statistically significant.

## Results

### Characteristics of the participants

**Fig. 1:**
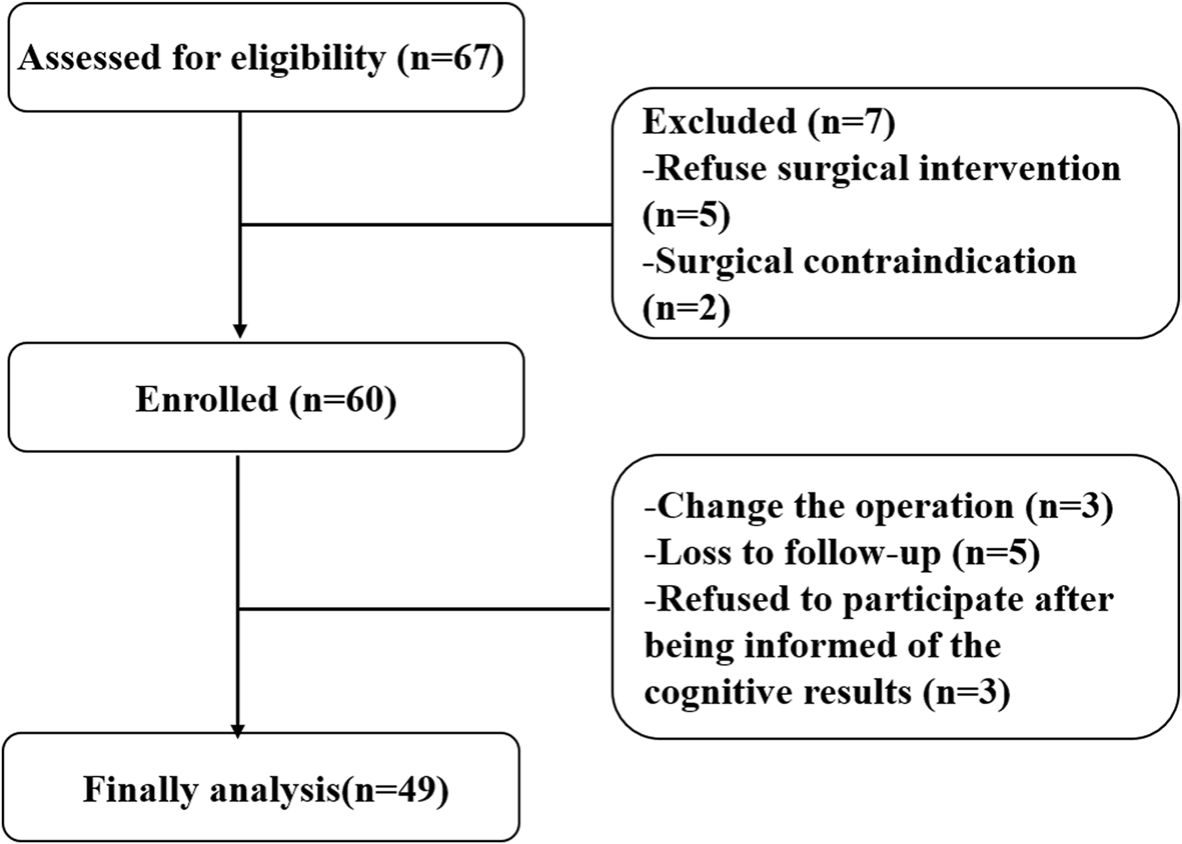
Participant flowchart

During the study period, 67 children were assessed for eligibility, but seven were excluded (refused surgery, *n* = 5; surgical contraindication, *n* = 2). Sixty children were enrolled, but 11 were excluded (change in surgery, *n* = 3; loss to follow-up, *n* = 5; refused to continue participation after being informed of the cognitive results, *n* = 3). Therefore, 49 patients were included in the analysis since they completed the 6-month follow-up and assessments (Fig. 1). Their characteristics are shown in Table 1. The children were 8.5 ± 1.9 years old before surgical intervention. Their body mass index increased after surgery (from 18.8 ± 4.9 to 19.3 ± 4.3 kg/m^2^, *P* = 0.008). The OAHI reduced after surgery [from 2.60 (1.60, 7.20) to 0.80 (0.50, 2.15), *P* < 0.001].

**Table 1:** Characteristics and growth and development of the patients

Parameters	Prior to surgery (*n*=49)	At 6 months after surgery (*n*=49)	*P*
Age (years)	8.5±1.9	9.2±1.8	<0.001
Weight (kg)	35.4±14.6	39.1±14.7	<0.001
Height (cm)	135.1±12.5	140.1±12.9	<0.001
Body mass index (kg/m^2^)	18.8±4.9	19.3±4.3	0.008
Z score	-0.35 (-0.79, 0.67)	-0.34 (-0.74, 0.64)	0.964
Obstructive apnea/hypopnea index	2.60 (1.60, 7.20)	0.80 (0.50, 2.15)	<0.001
Learning disabilities-Pupil Rating Scale			
Total	73.8±12.7	84.6±10.3	<0.001
Verbal	28.0±10.7	37.1±10.8	<0.001
Non-verbal	45.7±3.9	47.4±3.6	0.047

### Myklebust PRS

The total PRS score increased at 6 months after surgery (from 73.8 ± 12.7 to 84.6 ± 10.3, *P* < 0.001), including the verbal (from 28.0 ± 10.7 to 37.1 ± 10.8, *P* < 0.001) and non-verbal (from 45.7 ± 3.9 to 47.4 ± 3.6, *P* = 0.047) subscores (Table 1).

### Quality of life and anxiety

Table 2 shows that the total score and all aspects of the ISLQ, except anxiety experience (from 5.4 ± 1.9 to 5.7 ± 1.9, *P* = 0.185) and physical emotion (from 5.0 ± 1.8 to 5.5 ± 1.7, *P* = 0.078), were improved at 6 months after adenotonsillectomy (all *P* < 0.01). The SAS score also decreased significantly from 20.1 ± 10.0 to 12.8 ± 6.6 (*P* < 0.001).

**Table 2:** Changes in quality of life before and after surgery

		Prior to surgery (*n*=49)	At 6 months after surgery (n=49)	*P*
ISLQ	Family life	5.1±1.5	5.8±1.2	0.001
	Peer interaction	5.2±1.2	5.8±1.3	0.003
	School life	3.8±1.9	4.7±1.6	<0.001
	Living environment,	4.6±1.6	5.3±1.5	<0.001
	Self-awareness	4.6±1.7	5.7±1.8	<0.001
	Cognitive component	43.2±16.5	53.6±14.1	<0.001
	Depression experience	4.0±2.6	5.2±2.1	<0.001
	Anxiety experience	5.4±1.9	5.7±1.9	0.185
	Physical emotion	5.0±1.8	5.5±1.7	0.078
	Emotional component	48.7±19.9	55.1±19.2	0.001
	Total	41.2±15.7	53.3±13.4	<0.001
SAS		20.1±10.0	12.8±6.6	<0.001

*SAS* Zung Self-rating anxiety scale, *ISLQ* Inventory of subjective life quality

### Conners PSQ

The results of Conners PSQ are shown in Table 3. All six dimensions of the PSQ, including conduct (61.5 ± 15.4 vs. 50.8 ± 10.1), learning (61.3 ± 12.7 vs. 49.2 ± 10.7), somatopsychic disturbance (60.8 ± 18.3 vs. 49.8 ± 11.0), impulsivity and hyperactivity (58.9 ± 11.1 vs. 46.5 ± 9.1), anxiety (55.0 ± 9.9 vs. 49.9 ± 7.6), and hyperactivity index (60.3 ± 11.4 vs. 49.0 ± 8.9), as assessed by the legal guardians, significantly decreased after adenotonsillectomy (all *P* < 0.01). Furthermore, the proportions of abnormality also showed a significant decrease in all six dimensions, which was in line with the assessment of absolute value (all *P* < 0.05).

**Table 3:** Changes in Conners PSQ before and after surgery

Conners PSQ	Prior to surgery	At 6 months after surgery	*P*
Conduct	61.5±15.4	50.8±10.1	<0.001
Proportion of abnormality, n (%)	9 (18.4%)	2 (4.1%)	0.039
Learning	61.3±12.7	49.2±10.7	<0.001
Proportion of abnormality, n (%)	22 (44.9%)	6 (2.2%)	<0.001
Somatopsychic disturbance	60.8±18.3	49.8±11.0	<0.001
Proportion of abnormality, n (%)	11 (22.4%)	3 (6.1%)	0.008
Impulsivity and hyperactivity	58.9±11.1	46.5±9.1	<0.001
Proportion of abnormality, n (%)	30 (61.2%)	13 (26.5%)	<0.001
Anxiety	55.0±9.9	49.9±7.6	0.002
Proportion of abnormality, n (%)	17 (34.7%)	4 (8.2%)	0.001
Hyperactivity index	60.3±11.4	49.0±8.9	<0.001
Proportion of abnormality, n (%)	32 (65.3%)	14 (28.6%)	<0.001

*PSQ* Parent symptom questionnaire

### Attention ability

As displayed in Table 4, 21 children (42.9%) had various visual sense sustained attention abnormalities before surgery, while none had such abnormality at 6 months after surgery. The percentage of children who had severe, moderate, mild, and suspect visual sense sustained attention abnormality significantly decreased after surgical intervention (from 26 (53.1%) to 3 (6.1%), *P* < 0.001).

**Table 4:** Changes in attention ability before and after surgery

Sustained attention abnormality	Prior to surgery	At 6 months after surgery	*P*
Visual, n (%)			<0.001
Severe	16 (32.7%)	0	
Moderate	3 (6.1%)	0	
Mild	2 (4.1%)	0	
Suspect	5 (10.2%)	3 (6.1%)	
No	23 (46.9%)	46 (93.9%)	
Auditory, n (%)			<0.001
Severe	36 (73.5%)	12 (24.5%)	
Moderate	2 (4.1%)	0	
Mild	3 (6.1%)	9 (18.4%)	
Suspect	0	0	
No	8 (16.3%)	28 (57.1%)	
Both auditory and visual, n (%)			<0.001
Severe	19 (38.8%)	0	
Moderate	5 (10.2%)	1 (2.0%)	
Mild	5 (10.1%)	8 (16.3%)	
Suspect	3 (6.1%)	5 (10.2%)	
No	17 (34.7%)	35 (71.4%)	

Various degrees of auditory sustained attention abnormality were found in 41 (83.7%) children before surgery and 21 (42.9%) after surgery (*P* < 0.001). As for severe auditory sustained attention abnormality, there were 36 (73.5%) patients before surgery, while there were merely 12 (24.5%) after surgery.

Before surgery, 29 (59.2%) children had both visual and auditory sustained attention abnormalities, and this number decreased to nine (18.4%) after surgery (*P* < 0.001). All 19 patients with severe visual and auditory sustained attention abnormalities showed improvement of various degrees after surgical intervention.

## Discussion

Previous studies showed that children with OSA display several issues in physical health [12], development [14], and comprehensive cognitive functions [15–17, 19, 22–24]. Shpirer et al. [38] showed that hypoxemia correlated with attentional dysfunction in children with OSA. Adenotonsillectomy is the only definitive treatment for OSA [10, 11]. Still, previous studies did not comprehensively examine the effect of adenotonsillectomy on growth and development, emotional state, quality of life, attention ability, and cognitive dysfunction in children with OSA [27, 28]. Since adenotonsillectomy removes the excess tissues that cause upper airway obstruction, adenotonsillectomy could improve oxygenation during sleep, improve sleep quality, and perhaps mitigate cognitive dysfunction. This study suggests that timely adenotonsillectomy could improve the PRS, ISLQ, and PSQ and decrease anxiety and auditory/visual sustained attention abnormalities, suggesting positive impacts on the growth, development, quality of life, and comprehensive cognitive abilities of children with OSA. A study showed that the accuracy of assessing OSA impacts was higher when using multiple tools [39]. Conners PSQ is the most commonly used tool for children’s behavioral assessment, both at home and school [40]. Conner’s PSQ can be used clinically or in research. Ali et al. [41] showed that children with high-risk breathing disorders had higher scores on Conner’s PSQ.

There is no comprehensive study on the effect of adenotonsillectomy on children’s development, but some studies provide a few clues. A meta-analysis of three studies revealed moderate-quality evidence for improvements in quality of life, OSA symptoms, and behavior after adenotonsillectomy and high-quality evidence for lack of effect on neurocognitive performance [27]. Marcus et al. [11] reported that adenotonsillectomy improved the quality of life, impulsiveness, and emotional lability, with significant improvements in comprehensive cognitive abilities. Friedman et al. [42] showed that OSA-related cognitive dysfunction is reversible by adenotonsillectomy. Similarly, a pilot study by Goldstein et al. [43] showed that the scores of a standardized measure of behavior improved after adenotonsillectomy for upper airway obstruction. An early study by Gozal [44] showed that adenotonsillectomy improved the mean grades of children with sleep breathing disorders.

Regarding development, Nachalon et al. [14] reported improvements in growth, systemic inflammation, and calorie intake after adenotonsillectomy in children with OSA. Adenotonsillectomy could decrease the proportion of OSA children <25th percentile for weight, height, and BMI [45].

Furthermore, a previous study focused on comparing the quality of life and anxiety between the children with OSA and healthy children. It revealed the lower quality of life and higher anxiety in children with OSA [46], supported by a study showing higher anxiety and depression with sleep disturbances also in adults [47].

In the present study, adenotonsillectomy improved children’s growth with OSA and improved their quality of life and comprehensive cognitive abilities. This is supported by Wei et al. [48], who showed improvements in sleep quality and behavior after adenotonsillectomy. Of course, various scales and tools are available to assess the changes in growth and development, quality of life, behavior, and attention ability. The discrepancies among studies could be due, at least in part, to such differences. Nevertheless, large-scale multicenter trials should be performed to determine the effects of adenotonsillectomy in children with OSA in multiple aspects.

This study has limitations. This was a single-arm study, and the lack of a comparator group precludes a firm conclusion on the effect of adenotonsillectomy on the development of children. Besides, the sample size was small and from a single center. Future studies should address these issues and examine biochemical parameters like low-grade inflammation, immune system, and stress hormones.

## Conclusion

In conclusion, after adenotonsillectomy, the PRS, ISLQ, and PSQ improved, and anxiety and auditory/visual sustained attention abnormalities decreased, suggesting positive impacts on the growth, development, quality of life, and comprehensive cognitive abilities of children with OSA. The results could help improve the management of children with OSA.

## Data Availability

All data generated or analyzed during this study are included in this published article.

## Abbreviations

AASM: American Academy of Sleep Medicine
BMI: Body mass index
CPAP: Continuous positive airway pressure
CPT: Continuous Performance Task
ICSD-3: The third edition of the International Classification of Sleep Disorders
ISLQ: Inventory of subjective life quality
OAHI: Obstructive apnea/hypopnea index
OSA: Obstructive sleep apnea
PRS: Pupil rating scale
PSQ: Parent symptom questionnaire
SAS: Self-rating anxiety scale
